# Social Inequity and Structural Barriers to Completion of Ecological Momentary Assessments for Young Men Who Have Sex With Men and Trans Women Living With HIV in San Francisco

**DOI:** 10.2196/13241

**Published:** 2019-05-08

**Authors:** Caitlin M Turner, Sean Arayasirikul, Dillon Trujillo, Victory Lê, Erin C Wilson

**Affiliations:** 1 San Francisco Department of Public Health San Francisco, CA United States

**Keywords:** ecological momentary assessment, EMA, MSM, trans women, people living with HIV, feasibility, acceptability

## Abstract

**Background:**

Ecological momentary assessments (EMAs) administered via text messaging facilitate real-time data collection. With widespread cell phone access, EMAs are becoming more available to even the most disenfranchised communities, such as those living with HIV. However, structural barriers disproportionately burden young men who have sex with men (MSM) and trans women (TW) living with HIV and threaten participation in HIV research.

**Objective:**

We aim to identify structural barriers to completing EMA text surveys nested within a digital HIV care intervention for young MSM and TW living with HIV in San Francisco.

**Methods:**

A total of 10,800 EMA text messages were delivered daily over 90 days to 120 participants enrolled in the Health eNav intervention (2017-2018) at the San Francisco Department of Public Health. EMA surveys inquired about participants’ daily affect, sexual behaviors, substance use, and treatment adherence. Survey completion was calculated after 30, 60, and 90 days of follow-up. We described characteristics of nonstarters (those who provided less than four complete responses to the first seven EMA surveys) and analyzed structural correlates of days to first weeklong or more EMA survey noncompletion using multivariable Cox proportional hazards regression. Qualitative interviews were used to evaluate the acceptability of EMA surveys.

**Results:**

Participants completed 4384 of 10,800 (40.59%) EMA surveys. Completion of 70% or more of EMA surveys was attained by 56 of 120 participants (46.7%) at 30 days of follow-up, 40/120 (33.3%) at 60 days of follow-up, and 30/120 (25.0%) by the end of the 90-day study period. Twenty-eight participants (23.3%) were identified as nonstarters, and were more likely to be recently incarcerated (prevalence ratio [PR] 2.3, 95% CI 1.3-4.4), forego basic needs for HIV medications (PR 2.4, 95% CI 1.3-4.5), and be diagnosed with HIV in the last year (PR 2.2, 95% CI 1.1-4.1). Adjusting for nonstarters, young MSM and TW living in temporary/transitional housing (adjusted hazard ratio [aHR] 1.8, 95% CI 1.1-3.0), foregoing HIV medications to afford basic needs (aHR 1.7, 95% CI 1.1-2.7), and having less than a college education (aHR 3.5, 95% CI 1.4-9.0) had greater hazard of weeklong or more EMA survey noncompletion. Overall, there was high acceptability of the EMA surveys.

**Conclusions:**

Although access to and use of technology is increasingly ubiquitous, this analysis demonstrates persisting gaps in EMA completion by socioeconomic factors such as incarceration, education level, housing, and competing needs for young MSM and TW living with HIV in San Francisco. Moreover, those recently diagnosed with HIV were more likely to experience an immediate drop-off in completing EMA surveys. EMAs are feasible for individuals not experiencing social inequity and structural barriers. HIV prevention technologies addressing these barriers and leveraging similar methodology may prove effective for young MSM and TW living with HIV.

## Introduction

Ecological momentary assessments (EMAs) facilitate real-time data collection and may be administered through a number of technological platforms, such as handheld devices or mobile phones. Previous studies have used the terms “daily diaries” or “electronic diaries” interchangeably with EMAs; however, in this study, we will use only EMA for consistency. A range of research projects have used EMAs to improve data accuracy and to capture nuances in affect or mood [1], substance use [1-5], and, more recently, behaviors among persons living with HIV [5-8].

With ubiquitous mobile phone access, EMA and mobile interventions are available for communities with the greatest burden of HIV. As of 2014, more than 80% of young adults owned a mobile phone for information gathering and communication purposes [9]. A number of studies found that young men who have sex with men (MSM) use mobile devices to access sexual health information and consider mobile HIV interventions to be acceptable [10-12]. In a study of young, black MSM, researchers concluded that mobile HIV interventions may increase participation in HIV prevention and treatment [13]. One study of young MSM living with HIV showed high feasibility and acceptability of daily diaries measuring affect, substance use, and other risk behaviors [8]. Another study found acceptability of a peer-administered mobile health intervention among persons living with HIV who also had a history of substance use disorders and low adherence to antiretroviral treatment [5]. However, no studies exist that similarly examine the feasibility and acceptability of EMA among trans women (TW) living with HIV.

Currently, TW are among the highest risk groups for HIV with more than 49 times the odds of HIV infection than other reproductive age groups [14]. Efforts to collect data for this community could be vastly improved by using EMAs. However, to our knowledge, no quantitative studies of EMAs or other mobile health interventions exist for TW. Just as young MSM and other HIV risk groups consistently demonstrate high EMA compliance [1,2-4,8,15], TW living with HIV may similarly respond well to such a data collection method.

However, TW and MSM living with HIV face significant structural barriers which may threaten their participation in EMA research. For this analysis, we define structural barriers as institutional or social determinants of health (eg, housing instability, incarceration, and competing needs). Consistent with literature positing social factors as fundamental causes of HIV and other adverse outcomes [16,17], we approach EMA engagement in HIV interventions through a structural lens.

Seropositivity for HIV can be both an effect and a determinant of structural vulnerability and socioeconomic crises. Housing instability [18], racial/ethnic disparities [19], and poverty [20] promote HIV infection. People living with HIV experience barriers to economic opportunities due to HIV-related stigma and financial/logistical demands of accessing HIV-related care [21,22]. Competing needs (eg, prioritizing housing over health care) may put people at increased risk for HIV infection or may be exacerbated after people contract HIV [23]. When coupled with discrimination and adversity due to sexual or gender minority status, socioeconomic crises are exponentially worse for young MSM and TW living with HIV [24-26] compared to their nonminority counterparts. In one study of morbidity and mortality among persons living with HIV, homelessness and incarceration were associated with an increased mortality risk for MSM. These factors were not significantly associated for nonsexual minority participants and were not assessed for TW [27].

Few studies examine structural barriers to EMA completion. Two EMA feasibility studies among MSM showed that men without college degrees had lower EMA completion [2,4]. Prior research demonstrated inconsistent results regarding the relationship between race/ethnicity and EMA completion: two studies cite no differences in EMA completion by race/ethnicity [4,6], and one study showed lower EMA completion among MSM of color [2]. A study of risk behaviors among young people who inject drugs found that homeless participants had lower completion of EMAs [1]. Structural barriers to EMA completion have yet to be explored among MSM and TW living with HIV.

Individual characteristics such as recent HIV diagnosis and technological behaviors may also influence EMA study participation. We hypothesized that participants who generally exhibited high mobile technology interactions (ie, higher frequencies of sending, receiving, or checking text messages) would have greater EMA completion. We also hypothesized that newly diagnosed persons may be facing a unique set of experiences—higher depression risk [28] and HIV-related stigma—which may interfere with EMA completion. Finally, prior research has shown mixed results in terms of the relationship between age and EMA completion. A study among substance-using MSM showed lower completion among older MSM [2]; conversely, another study demonstrated that EMA was feasible and acceptable among older adults living with HIV [7]. However, technological behaviors, recent HIV diagnosis, and age have yet to be explored as possible facilitators or barriers to EMA completion among young TW and MSM living with HIV.

To address these research gaps, we conducted a mixed methods study of EMA feasibility and acceptability among young MSM and TW living with HIV who were enrolled in an HIV digital care navigation intervention in San Francisco. We also assessed EMA participation by structural and individual characteristics.

## Methods

### Study Design and Procedures

We analyzed data from Health eNav at the San Francisco Department of Public Health from 2017 to 2018. The Institutional Review Board at the University of California, San Francisco, approved the study procedures (IRB #16-19675). Health eNav is a digital HIV care navigation intervention that uses short message service (SMS) text messaging to improve outcomes along the HIV care continuum for young MSM and TW living with HIV.

On enrollment, participants completed EMAs that monitored their daily affect, sexual behaviors, substance use, and treatment adherence. This study focuses on the EMA component of the larger study. To assess feasibility, we analyzed EMA completion, hypothesizing that engagement in the EMA portion of the study would relate to engagement in the larger intervention. Characterizing EMA completion was also a necessary step for later incorporating it as an intervention exposure variable in future analyses. Participants were compensated US $1 for each completed EMA survey for up to US $90 over the EMA portion of the study. If participants completed more than 80% of their EMA surveys, they earned a bonus of US $100. Incentives were provided in the form of a gift card.

In addition to quantitatively analyzing feasibility, we assessed acceptability by conducting semistructured, in-depth interviews with a subsample of 16 participants 12 months after enrollment. Participants were purposively sampled to obtain diversity in race/ethnicity, gender identity, and level of engagement with the intervention. Participants were provided US $75 gift cards for their time. Interviews lasted 30 to 45 minutes and took place during a time that was most convenient for the participant. The interview guide was iterated to maximize coverage of participant experiences through theoretical sampling to reach theoretical saturation [29] and to address the following research question: “What factors impacted acceptability of EMA for young people living with HIV?” The interview guide assessed the following constructs: preferences, length, and individual- and health-related effects. Interviews were audio-recorded and transcribed verbatim. Transcriptions were randomly checked for quality and accuracy against original recordings. Qualitative interview data were coded and analyzed using grounded theory [29]. Two members of the research team independently coded qualitative data, line by line, and organized codes into categories to identify specific factors that shaped acceptability of EMAs for young people living with HIV.

### Participants

Eligible participants (1) identified as MSM or TW, (2) were between the ages of 18 and 34 years, (3) reported living in San Francisco, and (4) were newly diagnosed with HIV or not engaged/retained in care or not virally suppressed. Convenience sampling was used to recruit potential participants from five clinics and community-based organizations (CBOs) in San Francisco serving young people living with HIV. Posters, palm cards, and presentations (eg, in-service presentations, team huddles) were used to advertise study recruitment and to educate CBO and clinical staff. Staff referred potential participants to the study through phone/email communication and/or in-person meetings. Enrolled participants were also invited to refer peers from their social network.

Interested participants completed an in-person or telephone eligibility screening. If eligible, participants met with research staff at study offices located at the local health department to obtain informed consent and complete study enrollment activities. Overall, 171 people were screened for eligibility; of those, 140 were eligible, but 20 were lost to follow-up and did not enroll. In total, 120 participants were enrolled in the study. The majority of enrolled participants (90.8%, 109/120) were referred by clinics/CBOs and the rest (9.2%, 11/120) through peer referral. All 120 enrolled participants are included in this analysis.

### Measures and Variable Selection

For the EMA component of the study, participants were sent automated SMS text messages once per day at 8:00 am, 12:00 pm, or 8:00 pm for 90 consecutive days via mSurvey [30]. They were required to respond to EMA surveys within 24 hours. Participants could receive between 17 and 31 daily EMA texts depending on their responses. For example, if a participant responded “yes” to having sex within the last 24 hours, the participant would receive follow-up questions about whether condoms were used. Had the participant responded “no,” the participant would not receive subsequent questions about condom use. EMAs tended to take less than 5 minutes to complete each day. EMA completion was calculated day by day; EMA responses were considered incomplete if any or all of the 17 to 31 EMA texts received were skipped in a given day. Hypothesized barriers to EMA completion were collected via computer-assisted self-interviewing (CASI) surveys administered to participants at baseline and merged with EMA completion data.

#### Ecological Momentary Assessment Survey Completion

First, we computed cumulative EMA completion with the proportion of EMA surveys completed out of the total number of EMA surveys sent. We also calculated the percentage of participants who provided complete responses to 70% or more of the EMA surveys over 30 days of study follow-up. Health eNav followed participants for a relatively extended time period of 90 days; therefore, we also calculated EMA completion after 60 and 90 days.

We also characterized EMA completion in nontraditional yet clinically relevant ways. Hypothesizing that participants who failed to complete EMA surveys from the start of the study would be less likely to complete EMA surveys throughout the rest of the study, we defined “nonstarters” as those who did not complete a majority of the EMA surveys they received in the first week of the study (ie, those who provided less than four complete responses to the first seven EMA surveys sent).

We used survival analysis methods to calculate the number of study days that transpired before a participant failed to send complete responses to EMA surveys for at least seven consecutive days (ie, time to first weeklong or more EMA survey noncompletion), consistent with a previous study of EMA among substance-using MSM [2]. For this study, not completing EMA surveys for 7 or more days implied that no value was added by administering daily assessments over traditional weekly, biweekly, or monthly assessments. In addition, 1 week was a meaningful unit of time to characterize participants who may have had low completion in tandem with the overall digital navigation intervention. Classifying noncompletion in this way would allow us to later characterize participation in the overall digital HIV care intervention.

To visualize time to weeklong or more EMA survey noncompletion, we created an event plot and a Kaplan-Meier survival curve. Nonstarters were excluded from the Kaplan-Meier survival curve because we hypothesized that they represented a distinct outcome group.

#### Hypothesized Barriers to Ecological Momentary Assessment Survey Completion

Factors associated with time to first weeklong or more EMA survey noncompletion were selected a priori or were hypothesized as barriers to completion. Sociodemographics, such as age (in years), race/ethnicity (black/African American, Hispanic/Latinx, other/multiple, or white), and education level (less than high school, high school or GED, and at least some college), have been shown previously to influence EMA completion, with older participants, people of color, those with less than a college education, or those with lower income having lower EMA completion [2,4,6]. We also included housing status (living with a family member, friend, or partner who rents/owns a home; living in temporary/transitional housing; experiencing homelessness; or renting/owning a home) as a possible indicator of noncompletion, given previous literature citing homelessness as a barrier to participation [1]. Recent incarceration and competing needs (eg, foregoing HIV medications to afford basic needs such as food, housing, and/or clothing and vice versa) have been shown to exacerbate health outcomes for those living with HIV [23,27] but had yet to be explored as possible barriers to EMA participation.

We hypothesized that young TW and MSM who were recently diagnosed with HIV would face more challenges completing EMA surveys due to competing needs, stigma, and identity development related to seroconversion. Finally, we hypothesized that participants who had greater interactions with technology would have higher EMA completion. Therefore, we included frequency of sending/receiving or checking text messages on a mobile phone, categorized as once a day or less, several times a day, and several times an hour or all the time.

### Statistical and Qualitative Analyses

Analyses were conducted using Stata 14 software (StataCorp LP, College Station, TX, USA). Days to first weeklong or more EMA survey noncompletion, demographics, structural and socioeconomic factors, HIV diagnosis status, and technology behaviors were described for the entire sample (N=120) and bivariable Poisson binomial regression models [31] of these characteristics were analyzed for nonstarters compared to those who completed four or more EMA surveys in the first week of EMA surveys received.

Multivariable Cox proportional hazards models were used to calculate possible associations between the aforementioned sample characteristics and hazard of weeklong or more EMA survey noncompletion. Because nonstarters were analyzed as a separate group, we included nonstarter status as a covariate in multivariable survival analysis models of associations between structural barriers and noncompletion of EMA surveys. Proportional hazards assumptions were checked for each model. If any violations occurred, nonstarter status was interacted with time, and the models were rerun.

To assess acceptability of EMA, we conducted structured qualitative interviews postintervention of 16 participants. We used content analysis [32] to identify key themes, attitudes toward the EMA surveys, opinions about EMA survey length, and effect of the EMA surveys in general and on medical care.

## Results

Young MSM and TW living with HIV in Health eNav were racially/ethnically diverse, with a majority identifying as black or African American (22/120, 18.3%) or as Hispanic/Latinx (38/120, 31.7%). The average age was 27.8 (SD 4.1) years. Most participants lived in temporary/transitional housing (43/120, 35.8%) or rented/owned a home (39/120, 32.5%). A majority of participants earned US $1300 or less in monthly income (90/120, 75.0%), yet over half of the sample had an associate’s, technical/vocational, or bachelor’s degree or higher (68/120, 56.7%). Almost one in five participants reported being incarcerated in the last 6 months. Over a quarter of the sample had competing needs, either going without HIV medications because they needed money for basic needs or going without basic needs to afford HIV medications. Most participants (82/120, 68.3%) were diagnosed with HIV over a year prior to their baseline CASI assessment. A majority of participants reported that, in general, they frequently sent, received, or checked text messages on a mobile phone (Table 1).

**Table 1:** Sample characteristics for all participants and by nonstarter status (ie, completed less than four of the first seven EMA surveys) from the 2017-2018 Health eNav study (N=120).

Sample characteristics			All (N=120)	Completed ≥4 of first 7 EMA surveys (n=92)	Nonstarters (n=28)	PR^a^ (95% CI^b^)
Days to first weeklong or more EMA survey noncompletion, mean (SD)			41.87 (35.42)	51.35 (33.10)	10.71 (23.06)	0.95 (0.92-0.99)
**Demographics**						
	Age (years), mean (SD)		27.75 (4.07)	28.43 (5.88)	25.25 (2.22)	0.96 (0.88-1.04)
	**Gender identity, n (%)**					
		Trans woman	17 (14.2)	10 (11)	7 (25)	2.02 (1.02-4.02)
		Man	103 (85.8)	82 (89)	21 (75)	Ref^c^
	**Race/ethnicity, n (%)**					
		Black or African American	22 (18.3)	16 (17)	6 (21)	1.75 (0.60-5.04)
		Hispanic or Latinx	38 (31.7)	27 (29)	11 (39)	1.85 (0.72-4.79)
		Other^d^ or multiple	28 (23.3)	22 (24)	6 (21)	1.37 (0.47-1.03)
		White	32 (26.7)	27 (29)	5 (18)	Ref
**Structural factors**						
	**Housing status, n (%)**					
		Lives with a family member, friend, or partner who rents/owns a home	21 (17.5)	16 (18)	5 (18)	1.86 (0.60-5.72)
		Temporary/transitional housing^e^	43 (35.8)	30 (33)	13 (46)	2.36 (0.92-6.04)
		Homeless or shelter	17 (14.2)	12 (13)	5 (18)	2.29 (0.76-6.93)
		Rents/owns an apartment or house	39 (32.5)	34 (37)	5 (18)	Ref
	**Income in the last month (US$), n (%)**					
		$601-$1300	30 (25.0)	21 (23)	9 (32)	2.18 (0.75-6.32)
		$251-$600	30 (25.0)	24 (26)	6 (21)	1.45 (0.45-4.64)
		$0-$250	30 (25.0)	21 (23)	9 (32)	2.18 (0.75-6.32)
		≥$1301	29 (24.2)	25 (27)	4 (14)	Ref
	**Education, n (%)**					
		High school/GED	39 (32.5)	27 (29)	12 (43)	1.9 (0.93-3.91)
		Less than high school	13 (10.8)	8 (9)	5 (18)	2.38 (0.99-5.72)
		Some college or more	68 (56.7)	57 (62)	11 (39)	Ref
	**Incarceration in the last 6 months, n (%)**					
		Yes	23 (19.2)	13 (14)	10 (36)	2.34 (1.25-4.39)
		No	97 (80.8)	79 (86)	18 (64)	Ref
	**Competing needs, n (%)**					
		Went without HIV medications to have money for basic needs (eg, food, housing, and/or clothing)	38 (31.7)	66 (72)	16 (57)	1.62 (0.85-3.08)
		Went without basic needs (eg, food, housing, and/or clothing) to have money for HIV medications	32 (26.7)	26 (28)	12 (43)	2.38 (1.28-4.45)
**HIV diagnosis status, n (%)**						
	Diagnosed in the last year		38 (31.7)	24 (26)	14 (50)	2.16 (1.14-4.08)
	Diagnosed more than one year ago		82 (68.3)	68 (74)	14 (50)	Ref
**Behaviors toward technology, n (%)**						
	**Frequency of sending and receiving text messages on a mobile phone**					
		Several times a day	39 (32.5)	32 (35)	7 (25)	0.45 (0.18-1.12)
		Several times an hour or all the time	66 (55.0)	51 (55)	15 (54)	0.57 (0.26-1.22)
		Once a day or less	15 (12.5)	9 (10)	6 (21)	Ref
	**Frequency of checking for text messages on a mobile phone**					
		Several times a day	42 (35.0)	35 (38)	7 (25)	0.40 (0.15-1.04)
		Several times an hour or all the time	66 (55.0)	50 (54)	16 (57)	0.58 (0.26-1.29)
		Once a day or less	12 (10.0)	7 (8)	5 (18)	Ref

^a^PR: crude prevalence ratio from bivariable Poisson binomial regression models comparing nonstarters to those who completed four or more of the first seven EMA surveys received.

^b^CI: confidence interval.

^c^Ref: reference group.

^d^“Other” race/ethnicity included participants who identified as American Indian or Alaska Native (n=6) or Asian (n=7).

^e^Temporary/transitional housing included participants who lived in single room occupancy hotels, motels, boarding houses, halfway houses, drug treatment centers, independent living units, domestic violence shelters, battered persons’ shelters, or “safe houses.”

### Ecological Momentary Assessment Survey Completion

A total of 10,800 EMA surveys were sent to all 120 Health eNav participants over 90 days. Cumulatively, participants completed 4384 of 10,800 (40.59%) EMA surveys. At least 70% EMA completion was achieved by 56 of 120 participants (46.7%) by 30 days of follow-up, 40 of 120 participants (33.3%) by 60 days of follow-up, and 30 participants (25.0%) by 90 days of follow-up.

Almost one in four participants in Health eNav were nonstarters who completed less than four of the first seven EMA surveys they received. Nonstarters were more likely to have had less days to first weeklong or more EMA survey noncompletion (crude prevalence ratio [PR] 0.95, 95% confidence interval [CI] 0.92-0.99, *P*=.02), be TW (PR 2.02, 95% CI 1.02-4.02, *P*=.045), be incarcerated within the last 6 months (PR 2.34, 95% CI 1.25-4.39, *P*=.008), forego basic needs to afford HIV medications (PR 2.38, 95% CI 1.28-4.45, *P*=.006), or be diagnosed with HIV within the last year (PR 2.16, 95% CI 1.14-4.08, *P*=.02).

**Figure 1:**
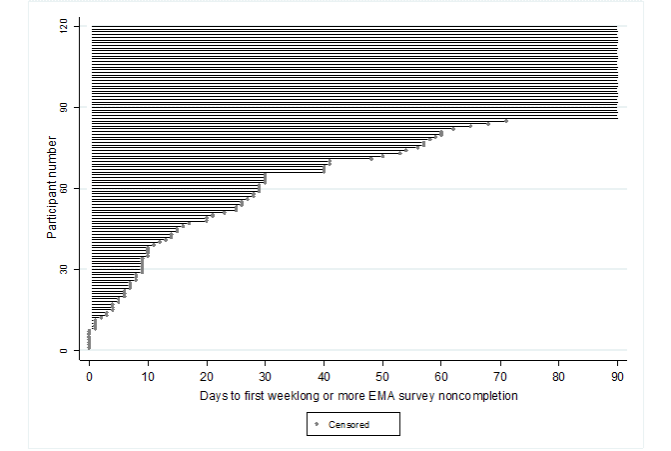
Event plot censoring the follow-up days that transpired before participants’ first experience of a weeklong or more ecological momentary assessment (EMA) survey noncompletion from the 2017-2018 Health eNav study (N=120).

**Figure 2:**
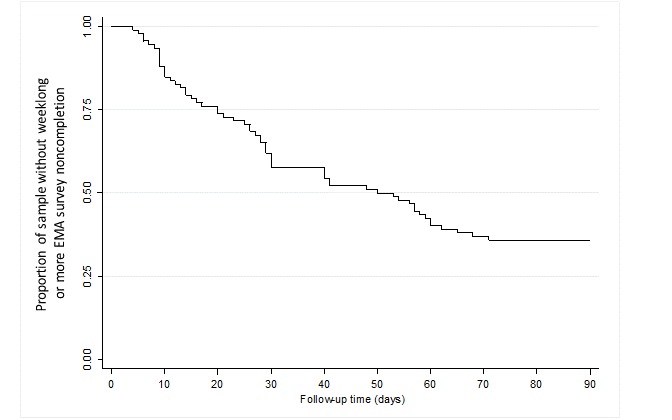
Kaplan-Meier survival curve visualizing the proportion of sample (excluding nonstarters, n=28) without a weeklong or more EMA survey noncompletion over the 90-day follow-up period from the 2017-2018 Health eNav study (n=92).

Days to weeklong or more EMA survey noncompletion for each participant are visualized in Figure 1. Figure 2 displays the proportion of the Health eNav sample (excluding nonstarters) who had not yet experienced a weeklong or more EMA survey noncompletion over the 90-day follow-up period.

Excluding nonstarters, the average time to weeklong or more EMA survey noncompletion was approximately 51 (SD 33) days. Of the 120 participants, 85 (70.8%) experienced a weeklong or more noncompletion; the average time to weeklong or more noncompletion was 22 (SD 20) days.

### Hypothesized Barriers to Ecological Momentary Assessment Survey Completion

Table 2 shows results from multivariable Cox proportional hazards models.

Adjusting for nonstarter status, we found that young MSM and TW living with HIV who lived in temporary/transitional housing (eg, single room occupancy hotels, motels, boarding houses, halfway houses, drug treatment centers) had higher hazards of weeklong or more EMA survey noncompletion compared to those who rented or owned a home (adjusted hazard ratio [aHR] 1.78, 95% CI 1.06-3.01, *P*=.03). Similarly, those who had a high school education compared to those with some college education or more (aHR 1.83, 95% CI 1.16-2.89, *P*=.01) and those who went without HIV medications to afford basic needs (aHR 1.71, 95% CI 1.09-2.71, *P*=.02) had a higher adjusted hazard of weeklong or more EMA noncompletion.

After testing proportional hazards assumptions and assessing variable time dependence using Therneau and Grambsch’s test of nonzero slopes [33], we concluded that nonstarter status should be interacted with time in our Cox proportional hazards models to assess the difference in hazards ratios by nonstarter status. Including the time-nonstarter status interaction, these post hoc survival analyses yielded similar conclusions as our original multivariable models for participants living in temporary/transitional housing (aHR 1.72, 95% CI 1.01-2.94, *P*=.047) and those who had a high school education (aHR 1.74, 95% CI 1.12-2.70, *P*=.01). However, competing needs (ie, foregoing HIV medications to afford food, housing, and/or clothing) was no longer significantly associated with greater hazard of weeklong or more EMA noncompletion (Table 2).

**Table 2:** Sample characteristics in relation to Cox proportional hazards of weeklong or more ecological momentary assessment survey noncompletion from the 2017-2018 Health eNav study (N=120).

Sample characteristics				With nonstarter status covariate, aHR^a^ (95% CI^b^)	With time-nonstarter status interaction, aHR (95% CI)
**Demographics**					
	Age			1.03 (0.96-1.09)	1.03 (0.97-1.09)
	**Gender identity**				
		Trans woman		1.60 (0.85-2.99)	1.41 (0.79-2.51)
		Man		Ref^c^	Ref
	**Race/ethnicity**				
		Black or African American		1.18 (0.65-2.12)	1.08 (0.58-2.01)
		Hispanic or Latinx		0.75 (0.42-1.36)	0.76 (0.43-1.36)
		Other or multiple		0.50 (0.23-1.09)	0.56 (0.27-1.16)
		White		Ref	Ref
**Structural factors**					
	**Housing status**				
		Lives with a family member, friend or partner who rents/owns a home		0.87 (0.38-1.97)	1.26 (0.63-2.50)
		Temporary/transitional housing		1.78 (1.06-3.01)	1.72 (1.01-2.94)
		Homeless or shelter		1.49 (0.73-3.04)	1.47 (0.71-3.06)
		Rents/owns an apartment or house		Ref	Ref
	**Income in the last month (US$)**				
		$601-$1300		1.16 (0.60-2.26)	1.34 (0.73-2.46)
		$251-$600		1.04 (0.57-1.91)	1.18 (0.68-2.06)
		$0-$250		1.13 (0.63-2.04)	1.15 (0.63-2.11)
		≥$1301		Ref	Ref
	**Education**				
		High school/GED		1.83 (1.16-2.89)	1.74 (1.12-2.70)
		Less than high school		1.12 (0.31-4.03)	1.55 (0.58-4.14)
		Some college or more		Ref	Ref
	**Incarceration in the last 6 months**				
		Yes		1.67 (0.98-2.85)	1.44 (0.84-2.47)
		No		Ref	Ref
	**Competing needs**				
		Went without HIV medications to have money for basic needs (eg, food, housing, and/or clothing)		1.71 (1.09-2.71)	1.46 (0.92-2.32)
		Went without basic needs (eg, food, housing, and/or clothing) to have money for HIV medications		1.58 (0.96-2.61)	1.29 (0.78-2.15)
**HIV diagnosis status**					
	Diagnosed in the last year			0.80 (0.46-1.39)	0.92 (0.56-1.51)
	Diagnosed prior to the last year			Ref	Ref
**Attitudes and behaviors toward technology**					
	**Frequency of sending and receiving text messages on a mobile phone**				
		Several times a day		0.70 (0.35-1.43)	0.79 (0.38-1.67)
		Several times an hour or all of the time		0.56 (0.29-1.06)	0.66 (0.35-1.25)
		Once a day or less		Ref	Ref
	**Frequency of checking for text messages on a mobile phone**				
		Several times a day		0.75 (0.33-1.69)	0.84 (0.37-1.92)
		Several times an hour or all of the time		0.61 (0.27-1.35)	0.72 (0.33-1.57)
		Once a day or less		Ref	Ref

^a^aHR: adjusted hazard ratio (adjusted for nonstarter status as a time-independent covariate or nonstarter status interacted with time).

^b^CI: confidence interval.

^c^Ref: reference group.

### Acceptability of Ecological Momentary Assessments Among Young People Living With HIV

Overall, most participants (14/16, 86%) who were qualitatively interviewed found the EMA surveys to be acceptable. Five of 16 (31%) participants cited that the surveys were easy to complete. Two participants reported that they enjoyed having the EMA surveys as part of their daily routine. Others (4/16, 25%) reported EMA participation was motivating, citing that completing the EMA surveys proved that they had the ability to commit to something and it was an opportunity to talk about uncomfortable topics such as sex or drugs. One participant said they “felt important” when asked questions about themselves on a daily basis. Few participants (3/16, 19%) felt unfavorably about the EMA surveys. For one participant, completing EMA surveys was an added burden and responding was a low priority in the context of other stressors in their life; however, they did find the remuneration motivating. One participant simply was uninterested, and another participant felt paranoid when they were in public and received a notification to begin their EMA survey. Regardless of whether participants found the EMA surveys to be favorable, some participants (4/16, 25%) found the surveys to be repetitive or redundant.

In terms of survey length and duration, only two (13%) felt that the surveys were too long or should be offered over a shorter duration of time. Most (9/16, 56%) suggested administering the surveys over a longer period of time to make a habit out of medication adherence and other self-monitoring skills they learned from the EMA surveys. They also felt that a longer EMA duration would allow them to see more positive or negative growth and become more accustomed to having a daily routine.

Many participants felt that the EMA surveys had a positive impact on their lives. Most participants (9/16, 56%) felt that EMA surveys improved self-monitoring of behaviors/mood and offered reminders to take HIV medications. Many (6/16, 38%) reported that the EMA surveys created a designated time for self-reflection regarding mood, habits related to substance use or sexual behaviors, and medication adherence. Specifically, one participant learned to ask themselves “What am I doing?” and “What can I change?” For one participant who regularly used substances, the EMA surveys were something to look forward to. For three other participants, the EMA surveys were similar to talking to a friend or having a loved one check up on them; EMA surveys increased their engagement in social relationships and community. In some cases (3/16, 19%), EMA surveys improved moods or habits. However, three participants (19%) reported no impact of the EMA surveys on their lives. Not all participants were in a stable situation to reap benefits from the surveys, citing housing (1/16, 6%), personal crises or stress (1/16, 6%), or phone turnover (2/16, 13%) as barriers.

Several participants cited health-related benefits from the EMA surveys. Seven (44%) felt that they received more insight into their physical and mental health status or cared more about their health. Half the sample felt they had more agency in managing their health care, citing confidence in changing doctors (1/16, 6%), adhering to medical appointments (3/16, 19%), knowing their rights as a patient (1/16, 6%), and being better able to report about their health and habits during doctor’s visits (1/16, 6%). One participant credited the EMA surveys for helping monitor their viral load and CD4 count.

## Discussion

Young MSM and TW living with HIV in San Francisco had moderate cumulative completion of EMA surveys (4384/10,800, 40.59%). Seventy percent or higher EMA completion was achieved by approximately half of the participants by 30 days of follow-up, and a quarter of participants by the end of the study (90 days). Results from this study suggest that although young MSM and TW living with HIV have access to mobile technology and they frequently send, check, and receive texts on their mobile phone, they still face substantial structural barriers to participating in EMA surveys administered via text messaging. Participants who experienced an earlier time to weeklong or more EMA survey noncompletion, were TW, were recently incarcerated, or who had competing needs were more likely to complete less than four of the first seven EMA surveys they received. A higher hazard of weeklong or more EMA survey noncompletion was experienced by participants who lived in temporary/transitional housing, had less than a college education, or went without HIV medications to afford basic needs. However, when we specified an interaction between time and nonstarter status, the association between competing needs and hazard of EMA noncompletion was no longer statistically significant. Qualitative interviews further cemented the role of structural barriers to EMA completion, with participants who reported low acceptability of the EMA surveys implicating housing instability and mobile phone turnover. Separately, in ad hoc analyses, we found that 30.0% (36/120) of the sample experienced no structural barriers (not less than college educated and not foregoing HIV medications for basic needs and not living in temporary/transitional housing). The average time to failure for these 36 individuals was 55.1 days, compared to 36.2 days for individuals who experienced at least one of these structural barriers (one-sided *t* test statistic: *t*_118_=2.75, *P*=.007).

Recent HIV diagnosis was also associated with greater likelihood of completing less than four of the first seven EMA surveys received. It could be that some young MSM and TW recently diagnosed with HIV were triggered by reminders of their new status during the course of the intervention, which interfered with their EMA participation. Although EMA surveys were not directly implicated in the following postintervention qualitative interview, one participant mentioned that participation was difficult because they did not want to be reminded about their new HIV diagnosis every day with the digital care navigation. Young MSM and TW recently diagnosed with HIV are an especially important group to engage in HIV interventions due to heightened susceptibility to depression and subsequent poor linkage to care [28]. This study provides novel, preliminary evidence that tailoring interventions to be sensitive to diagnosis timing may be critical for intervention participation among those who are newly diagnosed.

Our findings add novelty to the prior EMA literature. The cumulative completion we observed was lower than one study of young MSM living with HIV [8]; however, this could be a consequence of having a longer follow-up period compared to traditional EMA studies that span 1 week to 60 days [4,7,8]. Moreover, instead of excluding participants who failed to comply with EMA surveys during a calibration phase, we identified nonstarters (ie, those who completed less than four of the first seven EMA surveys they received) and analyzed them as a separate group. A number of EMA feasibility studies exclude participants during the calibration phase [8] or require that participants possess mobile phones with unlimited text plans [34], which inflates completion and excludes important information about participants who may experience additional challenges to EMA completion. This limits the generalizability of results. Had we removed nonstarters, similar to how participants were removed during calibration or screening phases in other studies, EMA completion would have increased by 10%.

In addition to identifying and analyzing nonstarters, we examined EMA noncompletion of 1 week or more. This enabled us to explore hypothesized correlates of the days that transpired before a weeklong or more noncompletion of EMA surveys. This time-to-event outcome offered greater nuance than a binary (low versus high) EMA completion outcome and subsequently greater precision in detecting significant associations. In using survival analysis methods, we were also able to account for censoring from participant dropout or from participants who had not yet experienced a weeklong or more noncompletion by the end of study follow-up.

The strengths of this analysis should be considered with its limitations in mind. Because this was the first study of EMA among young MSM and TW living with HIV, our analyses were largely exploratory, and findings should be interpreted as hypothesis-generating. Variable selection was based on prior literature and hypotheses; however, there were no prior EMA studies focusing on MSM and TW living with HIV. Although we utilized novel outcome classifications in our analyses, our time-to-event outcome definition poses another limitation to our study. Choosing a time-to-event of 1 week or greater could have been too short or too long to delineate meaningful windows of EMA noncompletion. However, having 1 week or longer of EMA noncompletion was a clinically relevant time unit. Several components of the Health eNav intervention necessitated weekly check-ins. Thus, failing to complete EMA surveys for 1 week or longer could signify noncompletion with the larger digital navigation intervention. In addition, if participants experienced noncompletion of a week or more, then there would be no added value to administering daily EMA surveys in lieu of weekly CASIs. A final limitation is that some point estimates were imprecise, probably due to the small sample size. However, relative to other EMA studies among MSM that have studied up to 70 participants for 30- or 60-day periods [3,8,15,34], this study consists of a larger number of participants and uses survival analysis methods with greater precision to detect associations. Additionally, this EMA study is embedded within a larger parent study that enrolled young people living with HIV who might benefit from an intervention aiming to improve linkage, engagement, and retention in HIV care. As a result, this sample may exhibit selection bias skewed toward the hardest-to-reach young persons living with HIV.

This analysis has a number of strengths and limitations that should be considered in subsequent research. Future studies should incorporate more complex causal pathways to examine correlates of EMA completion. The outcome classifications used in this analysis (eg, identifying nonstarters and days to first weeklong or more EMA survey noncompletion) represent a few of the ways in which EMA feasibility can be assessed; future research should similarly explore other ways of mapping noncompletion patterns depending on the study design and population. There are a number of additional structural and individual barriers to EMA completion beyond the scope of this study that could be examined, particularly for other HIV risk communities. If future studies find similar barriers to EMA completion for young sexual and gender minority groups living with HIV, this will lend credence to creating interventions that best address the unique needs of these communities. Our findings suggest that EMA is feasible and acceptable for individuals not experiencing social inequity and structural barriers, and that HIV prevention technologies addressing the previously mentioned barriers (eg, housing instability, incarceration, competing needs, educational constraints, and HIV diagnosis recency) and leveraging similar methodology may prove effective for young MSM and TW living with HIV.

Relatedly, these findings suggest that EMAs may be especially sensitive to experiences of structural barriers. A potential implication of these findings may be the uptake of EMAs in public health and clinical settings to better detect and identify the onset of structural barriers and social inequity in relation to real-time monitoring of engagement and retention in care, especially among vulnerable populations. Future studies should assess the intervention potential of EMAs in combination with other interventions at the individual level and systems level. EMA as a source of real-time feedback may inform personalization of public health service and health care utilization. Overall, these analyses lay the groundwork not only for future EMA studies among MSM and TW living with HIV, but also for characterizing EMA completion in other communities.

## Abbreviations

aHR: adjusted hazard ratio
CASI: computer-assisted self-interviewing
CI: confidence interval
EMA: ecological momentary assessment
MSM: men who have sex with men
PR: prevalence ratio
TW: trans women
